# Balancing pressure and support: family influences on female students’ educational choices and academic integrity in the UAE

**DOI:** 10.3389/fsoc.2026.1739469

**Published:** 2026-05-29

**Authors:** Maxime Jaffré, Shaikhah Basweidan, Tatiana Karabchuk

**Affiliations:** 1 Department of Government and Society, United Arab Emirates University (UAEU), Al Ain, United Arab Emirates; 2 Department of Cognitive Sciences, United Arab Emirates University (UAEU), Al Ain, United Arab Emirates

**Keywords:** academic integrity, Emirati women, ethical behavior, family influence, higher education, moral socialization, sustainability, UAE

## Abstract

This study examines how family influence shapes Emirati female students’ motivations, coping strategies, and ethical behavior within higher education. Drawing on Durkheim’s sociological perspective of education as moral socialization, it views the family as a meso-level institution that transmits collective consciences and sustains ethical responsibility across generations. While the United Arab Emirates (UAE) has achieved remarkable progress in women’s higher education, family expectations continue to define educational and professional choices, mediating the balance between empowerment and conformity. Using original survey data from 1,233 female students at the United Arab Emirates University (UAEU), the analysis explores how family motivations and pressures interact with students’ attitudes toward academic integrity and cheating behavior. Ordered probit regression models show that family influence is both enabling and constraining: family motivation is positively associated with diligence but may, under intense performance pressure, increase the likelihood of academic misconduct. Conversely, when family expectations are internalized as responsibility rather than anxiety, they strengthen students’ ethical self-discipline. The findings further reveal that attitudes toward integrity—particularly the conviction that cheating is never justified—mediate the relationship between family influence and academic behavior, confirming that ethical orientation, once internalized, guides conduct more strongly than external control. The study contributes to debates on ethics and sustainability in education by demonstrating that families remain key socializing institutions, shaping academic integrity through the transmission of ethical values rather than coercive authority. Promoting sustainable integrity in the UAE thus requires engaging both students and families in culturally grounded ethics education that aligns academic achievement with collective responsibility and social trust.

## Introduction

Families remain central to the organization of social life and the transmission of values across generations and societies. They shape not only patterns of care and identity but also pathways toward resilience, social cohesion, and sustainability. This view echoes Durkheim’s (1956) understanding of education as a social institution that transmits collective values and moral discipline necessary for social cohesion. As the primary environments in which individuals acquire cultural values, moral orientations, and future aspirations, families serve as both private networks of support and public institutions that mediate between individuals and society. Within this dual role, they are pivotal in shaping educational trajectories, instilling ethical sensibilities, and reinforcing intergenerational solidarity.

This study contributes to ongoing debates on families as agents of sustainability by exploring how familial motivations, expectations, and pressures influence female students’ educational choices, coping mechanisms, and academic integrity in the United Arab Emirates (UAE). It situates the analysis within a sociological framework that links family influence to the sustainability of cultural and ethical systems. In doing so, it views education not only as a means of individual advancement but also as a vehicle for transmitting collective values and ensuring intergenerational continuity. As Durkheim (1973) argued, moral education links individual development with the moral order of society, ensuring that social values endure through generations.

The UAE provides a particularly compelling context for this inquiry. Since the nation’s founding in 1971, rapid modernization, economic diversification, and global integration have reshaped the social landscape. Amid these transformations, family structures have retained remarkable strength, continuing to serve as moral and cultural anchors. This coexistence of global modernity and family-centered tradition produces a distinct social dynamic: women now significantly outnumber men in higher education, yet their academic and professional choices remain deeply embedded within familial expectations.

The expansion of Emirati women’s access to higher education is among the UAE’s most significant social transformation outcomes. According to the UAE Ministry of Foreign Affairs (2017), women now constitute the majority of students in higher education, with 77% of Emirati women enrolling in universities and making up 70% of all graduates in the UAE. However, this achievement coexists with persistent challenges. Despite high educational attainment, and comparatively high Emirati women’s labor-force participation unemployment rates among national women significantly exceeds that of men, especially among youth (18–34 y.o.)^1^ (World Bank, 2024). Scholars attribute this paradox to structural and cultural factors: while education is widely encouraged, families often influence or limit women’s career trajectories by prioritizing socially acceptable workplaces, typically within the public sector, near home, and in gender-segregated environments. As a result, education for many women becomes more a moral and social project shaped by family reputation, security, and collective advancement, rather than a path toward occupational independence (Rutledge and Madi, 2017; Labib, 2022).

Within this context, family influence operates as both enabling and restrictive. On one hand, families provide emotional, financial, and moral support, encouraging daughters to pursue higher education as an act of national and familial pride. On the other, they set boundaries on study fields, and career choices, particularly regarding mobility, gender mixing, and work environments. Education thus becomes a negotiated space between empowerment and conformity, where women’s ambitions must align with familial obligations and community values.

These dynamics extend beyond academic and career decisions to shape ethical orientations within higher education. International research shows that academic integrity, defined as the commitment to honesty, trust, fairness, and responsibility, depends not only on institutional rules but also on cultural and familial values (Akbar and Picard, 2020). In collectivist societies such as the UAE, where reputation and honor are deeply intertwined with family standing, students’ ethical decisions are often relational rather than individual. Academic success or failure reflects on the family as a whole, creating intense pressure to perform. Under such conditions, family expectations can inspire dedication and resilience but may also contribute to stress or rationalizations of unethical behavior when performance is threatened.

This study situates the question of academic integrity within this broader sociocultural field. Rather than treating academic misconduct (such as cheating or dishonesty) as isolated acts of individual failure, it examines them as products of complex social interactions where familial duty, institutional norms, and peer influence intersect. The family’s dual role as moral guide and source of pressure becomes particularly pronounced: it sustains motivation and ethical consciousness while simultaneously amplifying anxiety and the fear of failure. Understanding this tension is crucial to developing culturally grounded strategies for promoting academic integrity and ethical sustainability.

Focusing on undergraduate female students at the United Arab Emirates University (UAEU) provides a compelling lens through which to examine these complex social dynamics. First, Al Ain, where the university is located, is often described as the cultural heart of the UAE. Unlike the cosmopolitan hubs of Abu Dhabi or Dubai, Al Ain retains strong family-based social networks and a high proportion of Emirati nationals.^2^ This setting reinforces traditional values while offering a space for educational aspiration and intergenerational continuity. Second, UAEU is a favored destination for higher education among women, as it is perceived as a means of self-realization that simultaneously fulfills familial expectations and contributes to national progress.

This research aims to study the interplay of sustainability, ethics, and cultural resilience. The study argues that sustainability requires balance between ethical conduct and long-term resilience fostered by supportive family dynamics and excessive pressure that can erode integrity and trust at both personal and institutional levels. Thus, the research questions to answer here are as follows: How do family motivations, expectations, and pressures shape Emirati female students’ coping strategies when students face academic challenges? How do family-related motivations define students’ attitudes toward academic misconduct? Is there any relationship between family motivation and expectations on students’ academic integrity? And if yes, is it mediated by student’s attitudes toward cheating?

### Families as agents of educational and cultural sustainability in the UAE

While contemporary social structures have diversified, the family continues to serve as the primary environment in which individuals acquire not only cultural heritage but also moral orientations, resilience strategies, and ambitions for the future (Raevskikh et al., 2024). Scholars emphasize that families function simultaneously as private spaces of care and as public agents that mediate the relationship between individuals and broader societal challenges (Weiss-Laxer et al., 2020). In this dual role, they influence educational pathways in ways that are both enabling and constraining, situating personal advancement within a collective framework of duty, reciprocity, and solidarity.

Existing scholarship highlights the significance of family influence in shaping women’s educational and professional pathways in the UAE and wider Gulf region (Rutledge and Madi, 2017; Labib, 2022; Al-Jenaibi, 2015). Studies reveal that parents’ approval is often decisive in determining whether daughters pursue particular careers, accept positions outside their home cities, or pursue postgraduate opportunities abroad (Labib, 2022; Rutledge and Madi, 2017). Families are thus active negotiators of women’s aspirations, ensuring alignment with cultural expectations of honor, reputation, and responsibility. At the same time, families may encourage education as a means of enhancing social mobility and preparing younger generations for a knowledge-based economy (Forster et al., 2014). This dual role “enabler and gatekeeper” frames education as both a tool of empowerment and an arena of tension, where individual ambitions must be balanced against collective obligations.

Families provide the initial motivations for schooling, often framing education as a route not only to individual growth but also to collective advancement and intergenerational security (Eccles and Wigfield, 2020). This process reflects Durkheim’s conception of education as moral socialization, whereby family and school jointly prepare individuals to internalize the collective norms of society (Durkheim, 1973). Parents and extended kin serve as role models, transmitters of values, and gatekeepers of aspirations, encouraging perseverance in the face of challenges while shaping judgments about what constitutes success or failure (Rutledge and Madi, 2017; Labib, 2022; Eccles and Wigfield, 2020). Research from diverse cultural contexts highlights that children’s educational aspirations are profoundly shaped by family expectations, whether through direct encouragement, financial investment, or the moral obligation to honor intergenerational commitments (Eccles and Wigfield, 2020). Thus, the family’s role in education transcends mere support: it frames learning as a socially embedded process tied to ethical conduct, resilience, and long-term sustainability.

The UAE presents a unique case of rapid modernization alongside enduring family-centered traditions. Since its formation in 1971, the federation has pursued ambitious economic diversification, infrastructural development, and global integration, transforming from an oil-dependent economy to a regional hub of commerce, education, and innovation (Crupi and Schilirò, 2023). Amid these transformations, families have retained their role as core social units, shaping decisions about education, employment, and social mobility. This juxtaposition, between globalizing reforms and deeply rooted kinship norms, creates both opportunities and tensions for Emirati women navigating higher education and professional pathways.

Educational attainment among Emirati women is widely celebrated as one of the UAE’s major achievements. At higher education institutions such as UAEU, women account for over two-thirds of the student body, reflecting a strong cultural and policy emphasis on female participation in tertiary education (United Arab Emirates University, 2025). These patterns align with the UAE’s strategic visions, including the National Strategy for Higher Education 2030 and UAE Centennial 2071, which position education as central to building a knowledge economy and preparing youth for global competitiveness (UAE Ministry of Education, 2019; UAE Government, 2024).

**Figure 1:**
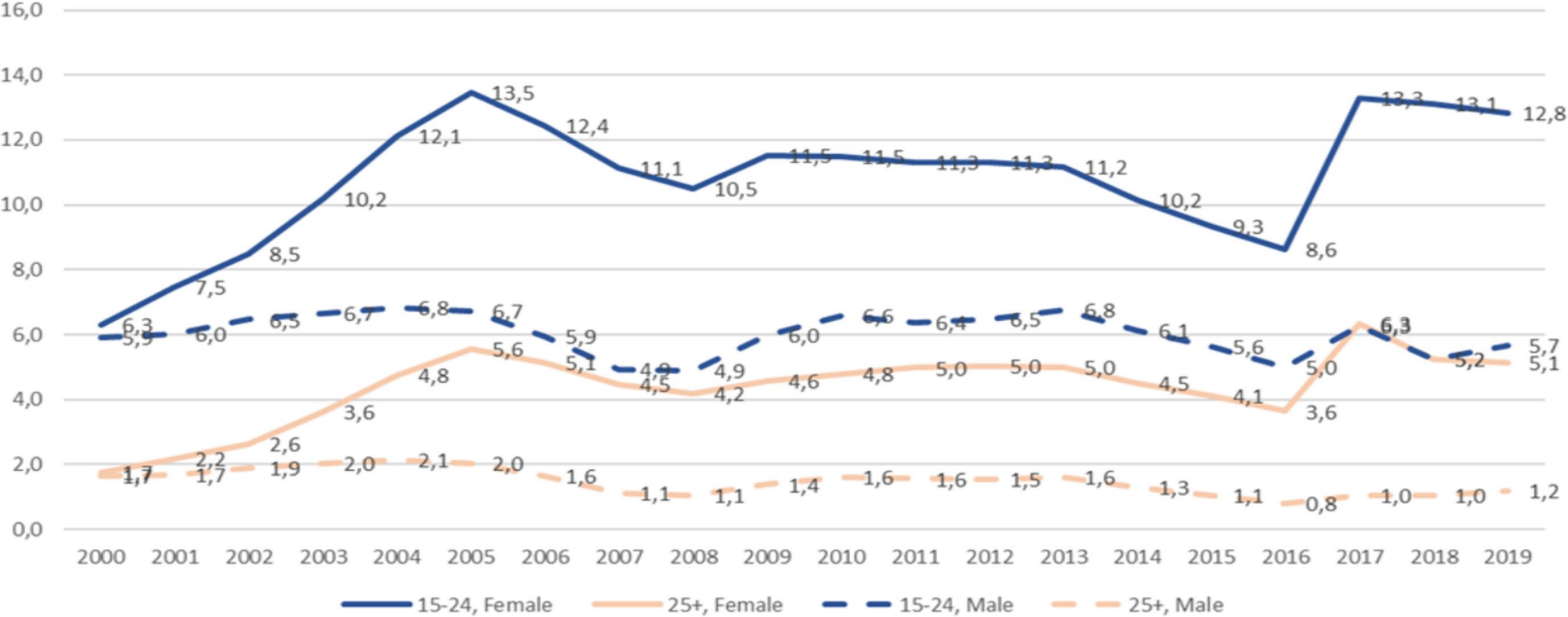
Dynamics of the unemployment rates by age and sex in the UAE, 2000–2019, UAE Federal Competitiveness and Statistics Centre (FCSC) data.

Despite this gender-reversed gap in higher education, Emirati women’s labor-force participation remains lower than men’s, and unemployment among national women is disproportionately high compared to male counterparts (Al-Jenaibi, 2015). For example, the unemployment rate among young Emirati females reached 12.8 percent in 2019, while the unemployment rate among males was only 5.7 percent (Figure 1, UAE Federal Competitiveness and Statistics Centre (FCSC), 2022). Research attributes this paradox partly to the segmentation of the UAE labor market, where nationals (especially women) prefer public sector roles due to their stability, shorter hours, and family-compatible conditions (Al-Jenaibi, 2015; Rutledge and Madi, 2017). Meanwhile, private sector jobs, often concentrated in globalized hubs such as Abu Dhabi and Dubai, may be deemed less desirable or less acceptable by families due to longer hours, mixed-gender environments, or commuting demands. These dynamics highlight the extent to which family expectations mediate the transition from higher education to employment, reinforcing the notion that families act as both enablers and gatekeepers in women’s professional trajectories (Rutledge and Madi, 2017; Labib, 2022).

Family influence in this context operates through two interrelated mechanisms. On the one hand, families invest heavily in daughters’ education, framing it as a means of securing future resilience, enhancing social reputation, and preparing women to contribute to society (Labib, 2022; Roman et al., 2025). On the other hand, families often impose restrictions on the types of careers, sectors, or geographic locations that are considered appropriate (Rutledge and Madi, 2017). This ambivalence is reflected in qualitative studies of Emirati women’s career choices, which show that parental approval is often a decisive factor in whether women pursue opportunities in globalized industries, accept positions in metropolitan areas, or continue postgraduate studies abroad (Forster et al., 2014). Education, therefore, is not an individual pursuit but a family-negotiated project, shaped by obligations of honor, reciprocity, and reputation.

These dynamics are particularly pronounced in the broader Gulf Cooperation Council (GCC) context, where sociologists have described families as “moral economies” that regulate the boundaries of acceptable female participation in education and work. In the UAE, this manifests in the coexistence of progressive state-led reforms promoting gender equality and enduring cultural frameworks that prioritize family oversight (Sinha et al., 2023). The state encourages women’s education as part of its modernization strategy, yet families mediate how this education is interpreted and applied, creating spaces of negotiation between aspirations for empowerment and concerns about tradition (Sinha et al., 2023; Rutledge and Madi, 2017).

This paradox situates Emirati women’s education at the intersection of modernization and cultural preservation. On the one hand, education is celebrated as a pathway to empowerment, equipping women with skills for participation in a globalized economy (Sinha et al., 2023). On the other hand, family-centered traditions continue to define the conditions under which this empowerment can be realized (Sinha et al., 2023; Rutledge and Madi, 2017). This tension resonates with broader sociological debates about the ambivalent role of families: they serve as carriers of resilience and sustainability but may also constrain individual agency when pressures to uphold honor or avoid shame outweigh aspirations for self-actualization (Rutledge and Madi, 2017; Labib, 2022; Roman et al., 2025).

Understanding this paradox is critical for examining academic integrity within Emirati higher education. While families provide strong motivational support for pursuing education, the pressures associated with maintaining family reputation and meeting expectations can also create vulnerabilities to maladaptive coping strategies.

These dynamics take on added significance when considering the issue of academic integrity. Global literature identifies cheating and dishonest practices as widespread challenges in higher education, often exacerbated by digital technologies and the pressures of competitive environments (Huang et al., 2025). In the UAE, researchers note distinctive cultural framings of academic dishonesty, where pressures to avoid family shame or to preserve household reputation may encourage some students to cut corners or justify misconduct (Aljanahi et al., 2024; Aljurf et al., 2019; Alsharefeen and Al Sayari, 2025). The rise of generative artificial intelligence has further complicated institutional responses, prompting universities to adopt both technological safeguards and traditional paper-based examinations as strategies to preserve integrity. Yet, little research has explored how family expectations intersect with these integrity challenges, particularly among Emirati female students whose educational journeys are deeply embedded in familial contexts.

This gap is particularly pressing given the sustainability lens through which education must now be viewed. Families are not only private units of support but also meso-level public agents that shape ethical orientations, resilience, and cultural continuity. Excessive pressure risks producing short-term coping strategies, such as cheating, that erode trust in institutions and undermine the sustainability of education as a system. Conversely, supportive family dynamics can foster resilience, responsibility, and a commitment to reciprocity, thereby contributing to the ethical sustainability of academic and societal institutions alike (Roman et al., 2025).

### Cheating, culture, family expectations, and integrity: the UAEU experience in global perspective

Academic integrity forms the moral and institutional backbone of higher education. It sustains trust in academic credentials and ensures that knowledge production reflects both intellectual rigor and ethical responsibility. Around the world, however, universities continue to confront rising levels of academic dishonesty, from plagiarism and collusion to the misuse of digital technologies that threaten the credibility of qualifications and the broader social mission of education (Karabchuk and Shomotova, 2025). Understanding why students cheat requires moving beyond simplistic notions of individual immorality to explore social, cultural, and institutional factors that normalize or discourage dishonest conduct (Aljurf et al., 2019). Following Durkheim’s (1956) perspective, ethical behavior must be viewed as a socially conditioned phenomenon shaped by collective conscience rather than individual pathology.

Globally, the expansion and digitization of higher education have reshaped academic competition. Students face unprecedented pressure to maintain high performance, often equating success with grades rather than learning (Eccles and Wigfield, 2020; Curtis and Clare, 2017; Forster et al., 2014). Studies across North America, Europe, and Asia show that many students rationalize cheating as a coping strategy for intense workloads and systemic competition (Bretag et al., 2018).

In the Arab world, academic dishonesty is similarly prevalent but uniquely embedded within collectivist cultural frameworks that emphasize social reputation, family honor, and reciprocity (Aljurf et al., 2019). Cheating is sometimes interpreted less as an individual ethical lapse and more as a pragmatic attempt to meet familial and social expectations (Aljurf et al., 2019). In such contexts, academic achievement is not merely personal; it reflects on the family’s status and the student’s obligation to uphold its reputation (Aljurf et al., 2019).

UAEU mirrors the tension between global academic standards and the moral expectations of a family-centered society. Faculty reports from UAEU and other Gulf institutions reveal persistent cases of exam misconduct, plagiarism, and unauthorized collaboration, often rationalized as “helping peers” or “avoiding shame” rather than intentional deceit (Aljanahi et al., 2024; Aljurf et al., 2019). Similar patterns appear in Saudi Arabia, where a cross-sectional study of 482 nursing students identified family-linked motivations, pressure to excel, fear of failure, and desire for social approval, as significant predictors of dishonest behavior (Alotaibi et al., 2024). In both contexts, families act as moral anchors and sources of pressure, framing educational success as filial duty and a marker of collective honor. Within this moral economy, cheating may emerge not from apathy but from devotion, a misplaced effort to fulfill familial and societal ideals of excellence (Farahat, 2022; Guerrero-Dib et al., 2020).

At the same time, the digital transformation of education has expanded both the possibilities and perceptions of dishonesty. The rise of online assessments, AI tools, and answer-sharing platforms has blurred moral boundaries, particularly among digital-native students who interpret “cheating” differently from their instructors (Pacino, 2021; Cotton et al., 2024). Instructors at UAEU and other UAE universities have observed what some describe informally as “cheating in style,” instances where technological creativity intersects with cultural norms. Reported cases include students using concealed earpieces, miniature Bluetooth transmitters^3^, and smartwatches hidden under sleeves or within traditional garments. While garments such as the abaya and sheyla symbolize modesty and identity rather than misconduct, their loose structure may inadvertently allow undetected communication, prompting gender-sensitive invigilation measures (Akbar and Picard, 2020). These localized practices underscore that cheating adapts to cultural and logistical contexts. As Bretag et al. (2018) argue, sustainable integrity cannot rely solely on surveillance or deterrence but must cultivate trust, moral reasoning, and culturally responsive ethics that align with both educational and societal values^4^.

Yet paradoxically, these same families transmit strong moral values that discourage dishonesty. For many students, family teachings about honesty, responsibility, and faith act as powerful deterrents against unethical behavior (Aljanahi et al., 2024). The coexistence of these opposing forces, like pressure and principle, illustrates the ambivalence of family influence.

Family remains one of the strongest influences on students’ values, goals, and choices in education. When families set clear expectations and actively motivate their children, they not only shape students’ academic ambitions but also their sense of honesty and responsibility. In this study, we argue that family support and expectations play a central role in shaping how students think and behave in relation to academic integrity and cheating.

High family pressure can force students into academic misconduct. However, we claim that this relationship is not direct or automatic, it is filtered through students’ own personal beliefs and attitudes toward integrity. Students who believe in working hard, find others’ cheating annoying, and see cheating as never justified are more likely to transform family expectations into genuine ethical behavior.

**Figure 2:**
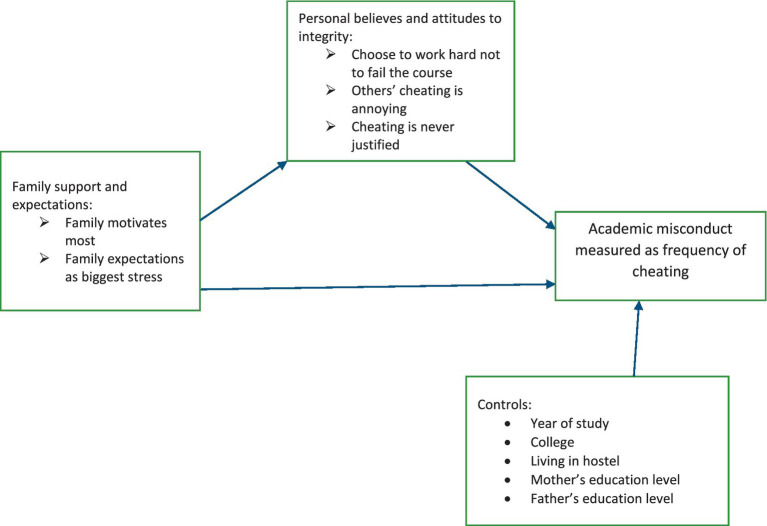
Conceptual framework.

In our empirical model below, we test if attitudes toward integrity serve as both a bridge and a filter between family influence and academic behavior (see Figure 2). As a mediator, these attitudes explain how family motivation leads to ethical conduct: strong family guidance nurtures integrity-oriented beliefs, which in turn reduces the likelihood of cheating. As a moderator, these attitudes may also strengthen or weaken the family’s impact. When students have firm ethical convictions, the positive influence of family expectations on honest behavior becomes stronger. But when students hold more tolerant views toward cheating, the family’s role may lose some of its power.

Hypotheses to test:

1. Stronger family support such as motivation is associated with lower frequency of cheating (H1a). At the same time family expectations perceived as biggest stress for the students would increase the frequency of academic misconduct (H1b).
2. More ethical values and positive attitudes toward integrity are associated with lower frequency of cheating (H2).
3. Attitudes toward integrity moderate the relationship between family expectations and the frequency of cheating, such that the negative association is stronger among students with higher integrity attitudes (H3).

### Data and methodology

#### Data description

This study draws upon an online survey conducted at UAEU, titled *Reasons for Pursuing Studies at the University and its Effects on Students’ Performance*. It was administered in 2024 and focused exclusively on the female student population. Ethical approval was obtained accordingly, and all participants were informed of the aims of the research, their right to withdraw at any time, and the strict guarantees of anonymity and confidentiality.

The survey was designed to capture, from an *emic* perspective, how female students articulate their motivations for studies, their academic challenges strategies, their attitudes to academic misconduct and their academic integrity. Questions were formulated to highlight not only individual decision-making but also the embeddedness of these decisions within broader family dynamics and cultural expectations. This design was intended to link micro-level behaviors such as cheating or perseverance with macro-level pressures stemming from family honor, cultural traditions, and societal expectations regarding women’s roles.

The questionnaire consisted of multiple-choice and scaled-response items. It included sections on: Coping strategies in the face of academic failure; Attitudes to academic misconduct and experiences with cheating; Perceptions of family and societal influence on their educational pathways.

The survey population consisted of undergraduate and postgraduate female students enrolled at UAEU. A total of 1,233 completed survey responses were collected, representing a substantial proportion of the university’s female student body. The survey female sample can be described as follows: the largest proportion of respondents were from the College of Humanities and Social Sciences (35.6%), followed by the College of Business and Economics (12.1%), the College of Engineering (11.3%), and the College of Information Technology (9.7%). Smaller shares represented the College of Science (10.0%), College of Education (6.8%), College of Law (5.8%), College of Medicine and Health Sciences (5.0%), and the College of Agriculture and Veterinary Medicine (3.2%), while the College of Graduate Studies accounted for 0.6% of the valid responses.

In terms of academic level, third-year (junior) students constituted the largest group (30.8%), followed by second-year (sophomore) students (29.3%), fourth-year (senior) students (19.2%), and first-year (freshman) students (16.3%). A smaller group of respondents (4.4%) reported studying for five or more years at UAEU.

#### Methodology description

To test the hypotheses an ordered probit regression modeling was applied since the dependent variable is measured as the frequency of cheating^5^ on the scale from 1 (never) to 4 (regularly). The main independent variables to test here were measured with the help of the following two questions:

1. What motivates you most to succeed academically? The answers such as ‘Giving back to my family and society’ or ‘Pressure from Family or Society’ were coded as 1 and all the other selected reasons as 0.
2. What is the biggest source of stress or anxiety for you? The answer of ‘High academic expectations from family or society’ was coded as 1 and all other answers as 0.

Among the moderating variables that were measuring students’ attitudes toward academic misconduct we identified the following three:

1. A student chooses ‘Work harder and seek help from a teacher/tutor’ answer (coded as 1) to the question ‘If you were at risk of failing a course, how would you most likely handle it?’ and all other answers (coded as 0) ‘Rely on my friends for support’ or ‘Find ways to minimize the impact of the failure on my grades’ or ‘Do whatever it takes to pass.’
2. Scale of the answers to the question on ‘How much does the behavior of other students cheating annoys you?’: It does not annoy me at all (1); It annoys me a little (2); It annoys me a lot (3).
3. Dummy variable for the choice that ‘cheating is never justified’ on the question ‘In which of the following situations do you feel cheating is justified?’

The ordered probit regression model also controls for socio-demographic background of the students such as student’s year of study, college, living in hostel, and father’s and mother’s education levels. These variables help isolate how family and personal beliefs contribute to shaping integrity-related behavior.

### Data analysis and findings

Since the research paper puts three research questions on the relationship between family support and expectations on the one hand and strategies to cope with academic challenges (1); attitudes to integrity and honesty (2) and academic misconduct (3) on the other hand, empirical analysis is implemented in the same structured plan.

First, the study provides descriptive statistical analysis on family support and expectations for the students and their strategies to cope with academic challenges, their beliefs and attitudes to integrity and academic misconduct. Second, the paper outlines the regression analysis outcomes to test the suggested relationship between the family support and expectations and students’ motivation to study and integrity compliance.

#### Factors motivating to succeed in studies

**Figure 3:**
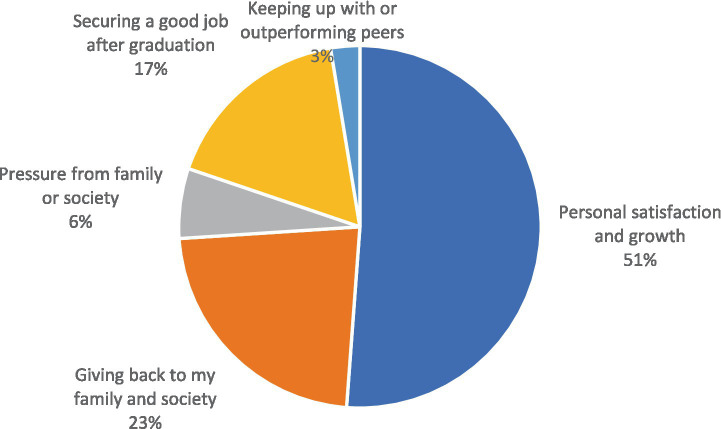
Primary motivation to succeed academically.

The survey reveals that female students at UAEU are primarily driven by intrinsic and value-based motivations rather than competition or purely instrumental aims. As shown in Figure 3, almost half of respondents’ (51 percent) identify personal satisfaction and growth as their chief motivation to succeed academically. A further 29 percent attribute their motivation to giving back to and meeting expectations from family or society, while 17 percent emphasize securing a good job as their goal. Only a small fraction (3 percent) are driven by peer comparison or competition.

The “Family Commitment Factor”, which merges responses originally coded as *giving back to family* and *pressure from family*, captures the dual nature of familial influence in Emirati society. It reflects a continuum between supportive duty and performance pressure, capturing how values of care, reciprocity, and honor shape women’s educational engagement. Culturally, this dynamic is rooted in the Emirati moral concept of *masʾūliyya*—a deeply embedded sense of responsibility toward one’s family and community (Hendrianto et al., 2021; Roman et al., 2025). Within this framework, succeeding academically is not simply a personal achievement but a moral act that fulfills intergenerational obligations. Yet, the same moral imperative that inspires perseverance can also generate anxiety, as students internalize the expectation to uphold family reputation and justify parental investment.

These findings confirm that, despite operating within a context of social expectation, Emirati female students conceptualize higher education as a pathway to self-development and moral responsibility rather than individual competition. The predominance of *personal satisfaction and growth* resonates with local values of *tahqiq aldhaat* (self-realization) aligned with *masʾūliyya* (responsibility) (Roman et al., 2025; Hendrianto et al., 2021). At the same time, the strength of the Family Commitment Factor underscores that the family remains an anchoring force—both a source of moral legitimacy and a potential site of pressure—shaping aspirations and framing women’s participation in higher education within a collective moral economy.

**Figure 4:**
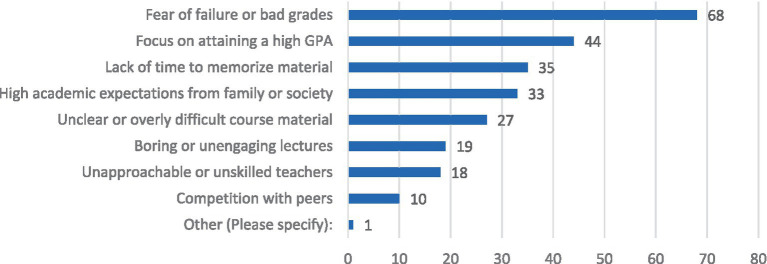
Distribution of answers to the question “What is the biggest source of stress or anxiety for you?” (multiple choice is possible), %.

Moreover, emotional weight of family expectations (33%) could be declared is quite high, taking fourth place after the fear of failure or receiving bad grades (68%), focus on attaining a high GPA (44%) and lack of time to memorize material (35%). Additional factors such as unclear or overly difficult course material (27%), boring or unengaging lectures (19%), and unapproachable or unskilled teachers (18%) suggest that classroom and teaching dynamics also contribute to students’ anxiety. Overall, the data show that students’ stress is largely shaped by fear of underperformance and the pressure to meet high academic and familial expectations (Figure 4).

#### Attitudes toward academic misconduct and academic integrity

**Figure 5:**
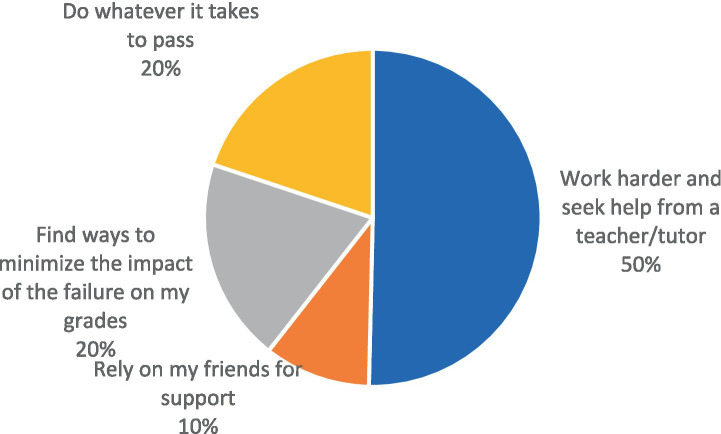
Distribution of answers to the question “If you were at risk of failing a course, how would you most likely handle it?”.

Among strategies to cope with academic challenges half of the respondents (50%) reported that they would work harder and seek help from a teacher or tutor, reflecting a constructive and integrity-based response aligned with internalized family expectations that emphasize effort, perseverance, and self-improvement (Figure 5). Another 20% indicated they would find ways to minimize the impact of failure on their grades, while an equal share (20%) said they would do whatever it takes to pass, a phrase that may imply a willingness to use questionable means when under pressure. A smaller proportion (10%) would rely on friends for emotional or practical support, suggesting that peer networks also play a modest role in how students manage academic stress. Taken together, these results suggest that while most students turn to legitimate strategies grounded in hard work and help-seeking, a notable minority may still be tempted to compromise integrity when the stakes are high. This finding reinforces the earlier model’s argument that attitudes toward academic integrity mediate how family expectations translate into actual behavior—strong family motivation may foster diligence for some students, yet for others, excessive pressure can blur ethical boundaries when facing the fear of failure.

**Figure 6:**
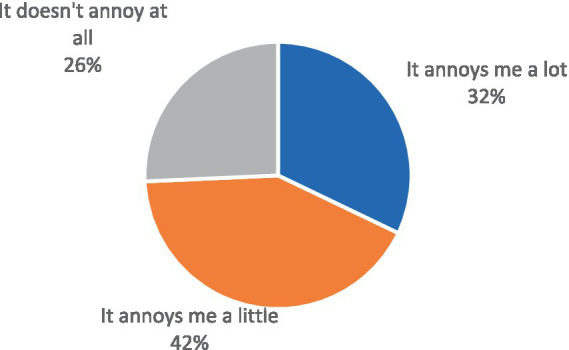
Distribution of answers to the question “How much does the behavior of other students cheating annoys you?”.

All in all, about 55% of the students believe that consequences of cheating are very serious at the university. It might explain their misconduct behavior. The Figure 6 below shows that most students experience at least some discomfort when others engage in cheating. About 42% said it annoys them a little, while 32% reported that it annoys them a lot, indicating that the majority express disapproval of dishonest behavior. Only 26% stated that it does not annoy them at all, suggesting a smaller group with more permissive or indifferent attitudes toward academic misconduct.

**Figure 7:**
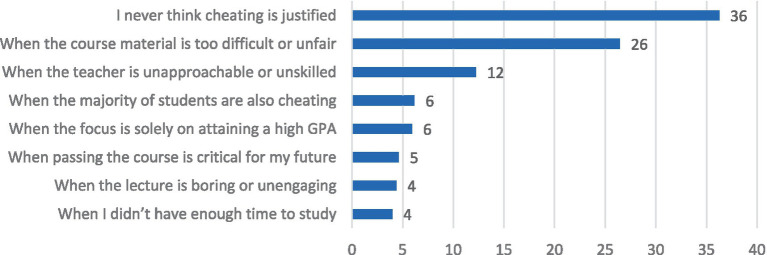
Distribution of answers to the question “In which of the following situations do you feel cheating is justified?”, %.

Figure 7 presents the distribution of answers to the question “In which of the following situations do you feel cheating is justified?” in percentages. The responses to this question reveal how students negotiate the boundaries of academic integrity in everyday life. More than a third (36%) stated that they never think cheating is justified, showing a clear moral conviction and a likely internalization of family and social values that emphasize honesty and effort. Yet, a considerable share (26%) admitted that cheating might be acceptable when the course material feels too difficult or unfair, while 12% pointed to situations where teachers are unapproachable or unskilled. These answers suggest that moral reasoning often operates within a context of perceived fairness and support: when students feel abandoned or overwhelmed, their ethical boundaries become more flexible. Smaller proportions justified cheating when others are also cheating (6%), when maintaining a high GPA or passing a course seems essential (5–6%), or when lectures are boring or time to study is limited (4%). Overall, these findings highlight that while many students hold integrity as a non-negotiable principle, others view it as conditional, meaning that it is something shaped by the pressures, inequalities, and expectations surrounding their academic lives.

**Figure 8:**
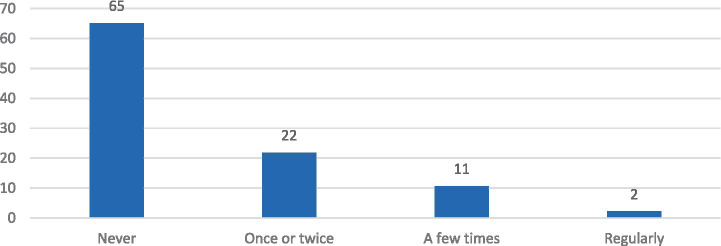
Distribution of answers to the question “How often have you engaged in cheating during your time at the university?”, %.

Finally, the survey allowed us to understand the scale of the academic misconduct behavior by self-reported answers to the question: “How often have you engaged in cheating during your time at the university?” (Figure 8). The majority (65%) stated that they have never cheated, suggesting that most students maintain a commitment to honesty, likely reinforced by personal values and family expectations around moral behavior and hard work. However, a notable 22% admitted cheating once or twice, and another 11% said they had done so a few times, indicating that occasional academic dishonesty is not uncommon and may emerge in response to situational pressures such as difficult coursework or fear of failure. Only 2% reported cheating regularly, showing that persistent misconduct is rare but still present. Overall, these findings reveal that while integrity remains the dominant norm, a meaningful minority of students navigate moments of moral compromise, reflecting the earlier model’s insight that attitudes toward academic integrity mediate how family expectations, academic stress, and individual choices interact in shaping actual behavior.

The next section of the results allows us to discuss the predictors of academic integrity, including the role of family motivation and expectation pressure.

#### Family motivation and expectations, attitudes to academic misconduct and academic integrity

This section of results provides the hypotheses’ tests outcomes. Firs we test the hypotheses H1a and H1b that family motivation and family stressful expectations predict frequency of cheating and H2 that attitudes toward academic misconduct also predict frequency of cheating.

For that we estimated a series of models, starting from zero-model, that included only control variables and then adding the tested independent variables one by one. Table 1 presents the results below. Overall, model fit improves substantially once moral attitude variables are added (Pseudo *R*^2^ rises from 0.008 to 0.086), showing that students’ ethical beliefs are stronger predictors of cheating behavior than demographic or family background factors alone.

**Table 1:** Ordered probit regression coefficients on frequency of cheating during university life.

Variable name	M0	M1	M2	M3	M4	M5
Family motivates most		0.155^*^		0.161^*^		0.111
		(2.06)		(2.14)		(1.44)
Family expectations are biggest stress			−0.174^*^	−0.180^*^		−0.180^*^
			(−2.30)	(−2.37)		(−2.30)
Choose to work hard					−0.137	−0.116
					(−1.88)	(−1.58)
Cheating is never justified					−0.882^***^	−0.891^***^
					(−10.51)	(−10.59)
Cheating is annoying					−0.257^***^	−0.247^***^
					(−5.30)	(−5.09)
Living in hostel	0.0379	0.0412	0.0429	0.0465	0.0875	0.0927
	(0.54)	(0.58)	(0.61)	(0.66)	(1.20)	(1.27)
Year of studies	0.0274	0.0331	0.0228	0.0285	0.0705^*^	0.0702^*^
	(0.81)	(0.97)	(0.67)	(0.84)	(2.02)	(2.00)
Mother’s education level	−0.0297	−0.0275	−0.0300	−0.0279	−0.0463	−0.0441
	(−0.80)	(−0.75)	(−0.81)	(−0.75)	(−1.23)	(−1.17)
Father’s education level	−0.0626	−0.0641^*^	−0.0622	−0.0636^*^	−0.0429	−0.0440
	(−1.96)	(−2.01)	(−1.94)	(−1.99)	(−1.31)	(−1.35)
_cons1	0.0683	0.126	0.000519	0.0583	−0.547^*^	−0.544^*^
	(0.35)	(0.63)	(0.00)	(0.29)	(−2.51)	(−2.44)
_cons2	0.816^***^	0.877^***^	0.751^***^	0.812^***^	0.286	0.293
	(4.10)	(4.32)	(3.73)	(3.96)	(1.29)	(1.29)
_cons3	1.722^***^	1.783^***^	1.659^***^	1.721^***^	1.267^***^	1.276^***^
	(8.12)	(8.21)	(7.77)	(7.86)	(5.49)	(5.37)
*N*	1,233	1,233	1,233	1,233	1,233	1,233
Pseudo *R*^2^	0.0078	0.0097	0.0102	0.0122	0.0831	0.0861

Models were also controlling for the college of studies. *t* statistics in parentheses. ^*^p < 0.05, ^**^p < 0.01, ^***^p < 0.001. Source: authors’ calculations based on the survey ‘Reasons for Pursuing Studies at the University and its Effects on Students’ Performance.’

Across models, several consistent patterns emerge that help to describe the complex relationship between family influences, integrity attitudes, and academic misconduct. In the baseline models M1 and M2, where we tested only for family variables inclusion, family motivation shows a modest significant positive association with cheating frequency, suggesting that students who view their families as their main source of academic motivation are more likely to engage in dishonest behavior. At the same time, if students report high stress because of family expectations that reduces the frequency of cheating. However, the effect weakens once integrity-related attitudes are included, indicating that the influence of family support is partly mediated by students’ internalized moral beliefs.

Students who believe cheating is never justified and those who find others’ cheating annoying show the largest negative associations with the frequency of cheating, underscoring the protective role of integrity-based values. The coefficient for working hard on academic challenges is also negative but only marginally significant, suggesting that while diligence matters, moral attitudes are a more decisive factor in preventing misconduct. Among control variables, year of study has a small positive effect (students in later years report slightly more cheating), while father’s education has a weak negative association.

To enhance interpretability, marginal effects from Model 5 were calculated and are shown in Table 2. These results express the impact of each variable on the probability of being in the lowest category of cheating (never cheating). The effects confirm the earlier interpretation: holding other factors constant, students who believe that cheating is never justified are approximately 29 percentage points more likely to report never cheating, while those who say others’ cheating is annoying are about eight percentage points more likely to do so. In contrast, students who experience family expectations as a major source of stress are around six percentage points more likely to report never cheating. The variables “family motivates most” and “working hard on academic challenges” show effects in the expected direction but do not reach statistical significance once moral attitudes are included. Taken together, these findings provide strong empirical support for the proposed conceptual model: family expectations and support influence academic integrity primarily through students’ internalized attitudes toward cheating, with stress amplifying risk and moral conviction offering the strongest protection.

**Table 2:** Marginal effects from ordered probit model M5.

Variable name	dy/dx	Std. err.	z	P > z	95% Conf.	Interval
Family motivates most to success in academia	−0.0363189	0.0252172	−1.44	0.150	−0.0857438	0.013106
Family expectations are biggest stress	0.0586256	0.0253509	2.31	0.021	0.0089387	0.1083125
Choose to work hard on academic challenge	0.0377341	0.0238008	1.59	0.113	−0.0089147	0.0843829
Cheating is never justified	0.2905905	0.024088	12.6	0.000	0.2433788	0.3378021
Cheating is annoying	0.0805133	0.0154408	5.21	0.000	0.0502499	0.1107766

Source: authors’ calculations based on the survey ‘Reasons for Pursuing Studies at the University and its Effects on Students’ Performance.’

To test the hypothesis H3 we estimated the same regression model but with interaction effects. Table 3 reports the results of the interaction models testing whether attitudes toward academic integrity moderate the relationship between family factors and the frequency of cheating. Specification (1) is the same model M5 without any interaction effects, specification 2 adds interaction effects with ‘family motivates most’, specification 3 adds interaction effects with ‘family expectations as biggest stress’.

**Table 3:** Ordered probit regression coefficients on frequency of cheating during university life with interaction effects.

Variable name	(1)	(2)	(3)
Family motivates most	0.111	−0.0769	0.114
	(1.44)	(−0.34)	(1.47)
Family expectations are biggest stress	−0.180^*^	−0.181^*^	0.0206
	(−2.30)	(−2.31)	(0.09)
Choose to work hard	−0.116	−0.0871	−0.107
	(−1.58)	(−0.99)	(−1.23)
Cheating is never justified	−0.891^***^	−0.885^***^	−0.866^***^
	(−10.59)	(−8.96)	(−8.85)
Cheating is annoying	−0.247^***^	−0.279^***^	−0.223^***^
	(−5.09)	(−4.91)	(−3.86)
Family motivates most*work hard		−0.0983	
		(−0.62)	
Family motivates most*cheating not justified		−0.0289	
		(−0.15)	
Family motivates most*cheating is annoying		0.120	
		(1.13)	
Family expectations*work hard			−0.0287
			(−0.18)
Family expectations*cheating not justified			−0.105
			(−0.54)
Family expectations*cheating is annoying			−0.0806
			(−0.75)
Living in Hostel	0.0927	0.0925	0.0953
	(1.27)	(1.26)	(1.30)
Year of studies	0.0702^*^	0.0725^*^	0.0709^*^
	(2.00)	(2.06)	(2.01)
Mother’s education level	−0.0441	−0.0442	−0.0434
	(−1.17)	(−1.17)	(−1.15)
Father’s education level	−0.0440	−0.0432	−0.0434
	(−1.35)	(−1.32)	(−1.32)
1 const	−0.544^*^	−0.586^**^	−0.480^*^
	(−2.44)	(−2.58)	(−2.09)
2 const	0.293	0.251	0.358
	(1.29)	(1.09)	(1.53)
3 const	1.276^***^	1.236^***^	1.340^***^
	(5.37)	(5.06)	(5.49)
*N*	1,233	1,233	1,233
Pseudo *R*^2^	0.0861	0.0867	0.0866

*t* statistics in parentheses. ^*^p < 0.05, ^**^p < 0.01, ^***^p < 0.001. Source: authors’ calculations based on the survey ‘Reasons for Pursuing Studies at the University and its Effects on Students’ Performance.’

Across all specifications, the main effects remain consistent with previous findings: students who believe that cheating is never justified and who are annoyed by others’ cheating are significantly less likely to report engaging in academic misconduct. The inclusion of interaction terms, however, provides a more nuanced picture of how family influences operate through students’ moral orientations. The results show that none of the interaction terms between family motivation and the three attitudinal variables (choosing to work hard, believing cheating is unjustified, and being annoyed by others’ cheating) reach statistical significance. Similarly, the interactions between family expectations as a source of stress and these attitudinal measures are non-significant.

These outcomes suggest that while family support and expectations each predict students’ ethical behavior, the strength of these effects does not appear to depend on students’ attitudes toward integrity. In other words, attitudes toward academic misconduct serve primarily as mediating rather than moderating mechanisms in this context: they help explain how family influences shape behavior but do not substantially alter how strongly those family influences matter. The relatively stable coefficients and unchanged pseudo-*R*^2^ values across the specifications further support this interpretation, indicating that the interaction effects add little explanatory power beyond the main relationships already established in the previous model.

These findings align closely with the conceptual model proposed earlier, which positioned attitudes toward academic misconduct as the central psychological mechanism linking family influence on students’ ethical behavior. The absence of significant moderation effects suggests that family expectations and motivation do not become more or less effective depending on students’ integrity attitudes; rather, those attitudes themselves are shaped by family environments and internalized through early socialization.

In this sense, the family functions as a moralizing institution, transmitting values that students carry into academic life. When those values become internalized as personal beliefs, such as the conviction that cheating is never justified, they operate as mediators that explain the pathway from family expectations to behavior, rather than as conditions that amplify or weaken it. This pattern reinforces a sociological understanding of integrity as rooted in social and familial moralization processes rather than situational or attitudinal contingencies. Families that combine high expectations with emotional support appear to foster ethical self-regulation, whereas those that impose pressure without fostering internalized integrity norms may inadvertently increase students’ stress and susceptibility to misconduct.

## Discussion

The findings confirm that families remain the primary source of motivation and ethical orientation for Emirati female students. Yet their influence is ambivalent—at once nurturing ethical behavior and creating pressure that can undermine it. Family thus shapes academic conduct not through direct control but through the ethical habits and beliefs it instills. This finding resonates with Durkheim’s (1973) notion that moral discipline originates in social life itself, with the family serving as the first environment of ethical education.

Consistent with earlier research (Weiss-Laxer et al., 2020; Roman et al., 2025), the Emirati family functions as a meso-level socializing institution transmitting values of honesty, perseverance, and *masʾūliyya* (responsibility). For many students, academic success represents a form of moral duty that reflects family honor and national pride (Rutledge and Madi, 2017; Labib, 2022). Findings from the survey “Reasons for Pursuing Studies at the University and its Effects on Students’ Performance” show that this framing motivates diligence but can also intensify anxiety. Regression results indicate that students primarily motivated by family expectations are slightly more likely to report cheating—suggesting that when success becomes tied to collective reputation, pressure may blur ethical boundaries.

Conversely, students who perceive family expectations as a major source of stress are *less* likely to cheat. This suggests that pressure framed as responsibility can reinforce ethical discipline. What matters is not the intensity of family expectations but how students internalize them. When familial demands are understood as expressions of trust and accountability rather than fear, they encourage persistence and integrity. This dynamic echoes Eccles and Wigfield’s (2020) expectancy-value theory, where achievement depends on the alignment between personal goals and socially shared values.

The study provides strong evidence that internal attitudes toward ethical behavior mediate the relationship between family influence and academic conduct. Students who believe cheating is never justified or who feel disturbed by others’ dishonesty are far less likely to engage in misconduct. These convictions—more than demographic or institutional factors—constitute the most effective safeguard of integrity. The absence of significant interaction effects indicates that ethical attitudes do not simply moderate family influence; they embody it. Once these values are internalized, they guide students’ behavior autonomously. This supports the view that ethical integrity stems from socialization within the family rather than from external surveillance or sanctions (Guerrero-Dib et al., 2020).

Emirati women’s educational paths unfold at the intersection of empowerment and obligation. Education is simultaneously a vehicle for self-realization and a means of fulfilling family and national expectations (Sinha et al., 2023; Al-Jenaibi, 2015). Within such a relational framework, acts of academic dishonesty may occasionally be rationalized as serving family or peer solidarity rather than individual gain (Aljurf et al., 2019; Akbar and Picard, 2020). Understanding integrity in this collective cultural setting is crucial: lapses in ethical behavior may reflect competing loyalties between institutional standards and communal ideals of responsibility.

Promoting academic integrity in the UAE therefore requires dialogue between families and universities rather than purely punitive measures. As Bretag et al. (2018) emphasize, sustainable integrity arises from transparency, fairness, and engagement. Embedding ethics education in culturally resonant frameworks—linking honesty to traditional and Islamic heritage principles such as *sidq* (truthfulness) and *Amanah* (trust)—can help students reinterpret family expectations as calls to ethical excellence. Parallel initiatives that include parents could encourage them to balance ambition with emotional support, reinforcing students’ confidence to achieve through honest effort.

Viewed through a sustainability lens, ethical behavior forms part of the social capital that upholds trust in educational institutions. Families that combine care with clear ethical guidance contribute to the sustainability of academic systems; those that impose performance pressure without open communication risk undermining it. Achieving balance between encouragement and accountability is therefore central to sustaining both integrity and intergenerational trust.

In summary, Emirati families exert a profound yet indirect influence on students’ honesty and academic commitment. Their impact operates through the internalization of moral beliefs rather than through coercion. When family expectations are translated into ethical purpose, they foster resilience and integrity; when they generate anxiety detached from ethical grounding, they may lead to misconduct. Sustainable education in the UAE must therefore nurture this equilibrium by aligning family, faith, and institutional ethics to ensure that achievement and honesty advance together.

## Conclusion

This paper examined the role of the family in shaping students’ academic integrity within the UAE context. Academic integrity in the UAE is not merely an institutional concern; it reflects how societies transmit values and ethical behavior across generations. This insight parallels Durkheim’s (1956) argument that education perpetuates the collective conscience by embedding shared norms and values within the individual. Recognizing the interplay of family pressure, technological change, and cultural honor allows educators and policymakers to design strategies that nurture both excellence and ethics, ensuring that academic success remains consistent with the enduring principles of honesty and sustainability.

The findings provide partial support for the first set of hypotheses (H1a–H1b). In the early models, family motivation showed a modest positive association with the frequency of cheating, suggesting that students strongly driven by family encouragement may also experience heightened performance pressure that can, in some cases, lead to dishonest coping strategies. Conversely, family expectations perceived as stressful were negatively associated with cheating, implying that when pressure is internalized as responsibility rather than anxiety, it can reinforce self-discipline and integrity. However, these family effects weaken once integrity-related attitudes are introduced, supporting the interpretation that family influence operates primarily through the internalization of ethical beliefs rather than direct behavioral control.

The second hypothesis (H2) is strongly supported. Students who believe that cheating is never justified and who express annoyance when others cheat are significantly less likely to engage in academic misconduct. These integrity-based attitudes serve as robust protective factors, while diligence (“choosing to work hard”) plays a secondary but reinforcing role.

The final analysis tested the moderation hypothesis (H3) by introducing interaction terms between family variables and attitudinal factors. None of these interaction effects reached statistical significance, indicating that attitudes toward integrity do not strengthen or weaken the relationship between family influence and cheating. Instead, they act as mediating mechanisms that explain how family norms and expectations are translated into ethical reasoning and academic behavior. This finding aligns with sociological understandings of ethical socialization: family environments cultivate ethical orientations that guide students’ actions, but once internalized, these orientations operate independently of situational variations in family support or pressure.

Overall, the results reinforce the central argument of this study: families remain key socializing institutions that shape academic integrity not through direct control but through the development of enduring ethical attitudes. Students who internalize family-based ethical norms demonstrate stronger resistance to misconduct, whereas performance-driven motivation without ethical grounding may inadvertently increase the temptation to cheat. Sustaining academic integrity thus requires both supportive family environments and the cultivation of strong personal convictions that reject dishonest behavior.

Integrity education in the UAE must therefore acknowledge this duality, encouraging students to reinterpret familial duty as a commitment to ethical excellence rather than mere performance. Addressing academic dishonesty in Gulf higher education demands a multi-layered approach that integrates enforcement, ethics education, and family engagement. Studies from Saudi Arabia (Alotaibi et al., 2024) show that faculty vigilance and clear institutional communication reduce permissiveness toward cheating. Similarly, UAE universities increasingly emphasize integrity charters and orientation programs that frame honesty as part of national and religious identity (Akbar and Picard, 2020; Alsharefeen and Al Sayari, 2025).

However, enforcement alone cannot sustain integrity. Bretag et al. (2018) advocate a proactive approach based on transparency, fairness, and dialogue between educators and students. Integrating discussions of ethics in curricula—through reflective writing, mentorship, and real-world case studies—can help students connect academic rules with lived ethical frameworks. In the UAE, linking integrity to traditional and Islamic heritage principles of honesty (*sidq*), trust (*amanah*), and accountability (*masʾūliyya*) offers culturally resonant pathways for strengthening ethical awareness.

Ultimately, academic integrity in the UAE reflects the broader transmission of ethical and heritage values across generations (Raevskikh et al., 2024). By acknowledging the intersections of family influence, technological change, and cultural expectations, educational institutions can sustain an environment where achievement and ethical behavior progress hand in hand in the pursuit of responsible, value-based excellence.

## Data Availability

The raw data supporting the conclusions of this article will be made available by the authors, without undue reservation.

## Footnotes

1 Labor force participation rate, female (% of female population ages 15+) – United Arab Emirates [Data set] (World Bank, 2024). World Development Indicators. https://data.worldbank.org/indicator/SL.TLF.ACTI.FE.ZS?locations=AE

2 According to the most reliable available estimates, Al Ain has one of the highest proportions of Emirati nationals in the UAE—about 30.8% of its population, whereas in the UAE overall, Emiratis constitute around 10–12% of the total population: https://worldpopulationreview.com/countries/united-arab-emirates. Official federal census data have not been published by the UAE government since 2005.

3 Monorean’s invisible earpiece systems: https://www.monorean.com/en

4 *Awareness reel on exam misconduct* [Instagram video]. United Arab Emirates University (2025) (UAEU). Instagram. https://www.instagram.com/reel/DG0FPfbo1e0/?igsh=anQ2a3ZobjhzenR4

5 How often have you engaged in cheating during your time at the university? 1 – Never; 2 – Once or Twice; 3 – A few times; 4 – Regularly.
